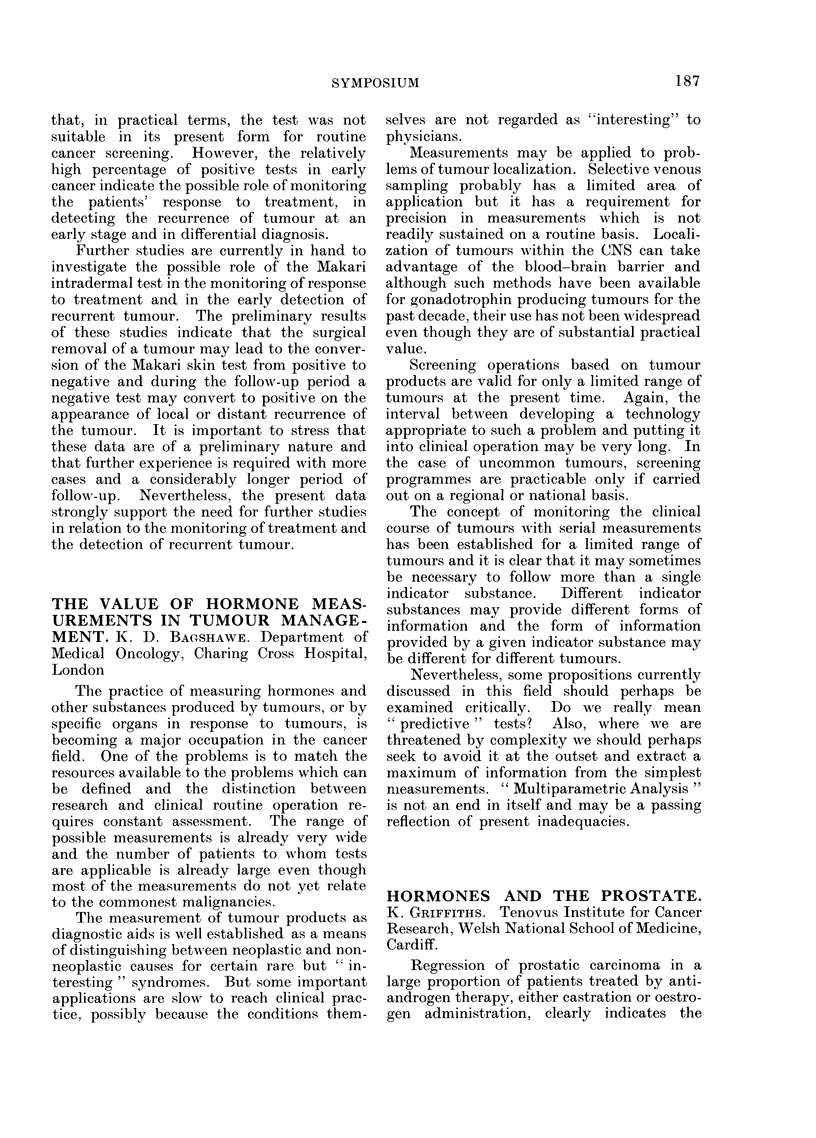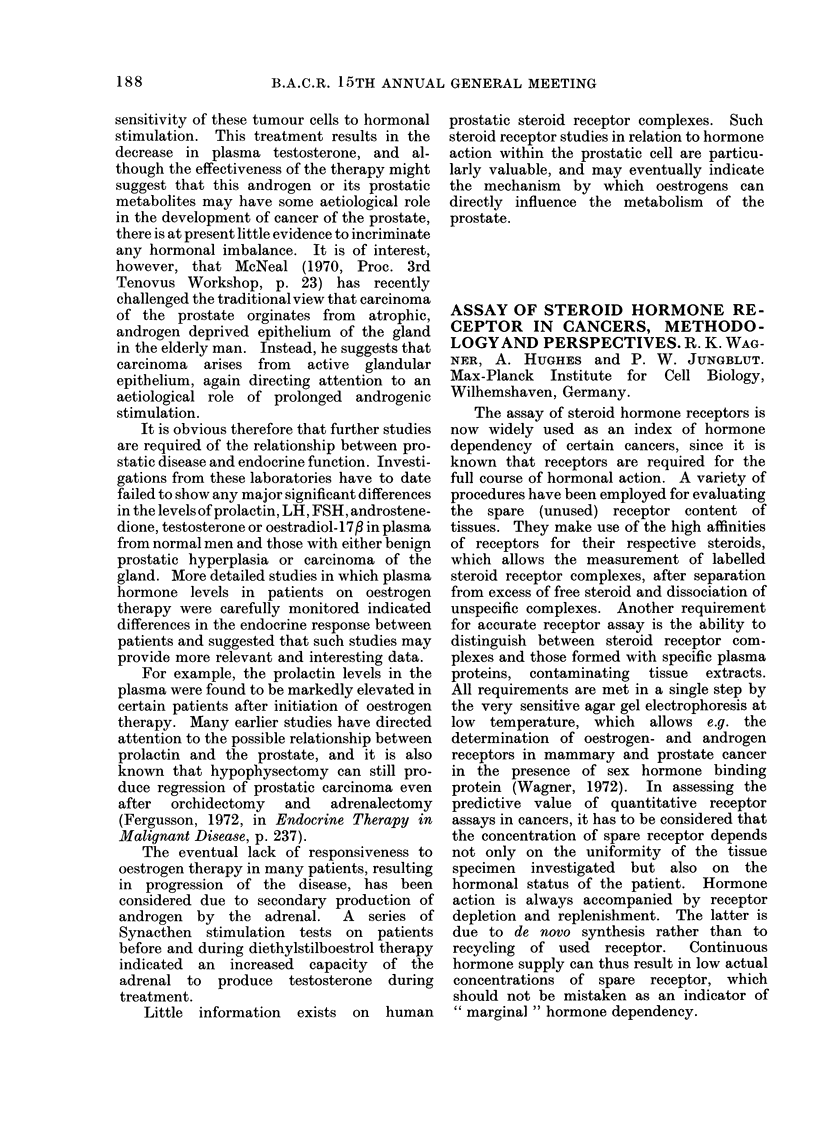# Proceedings: Hormones and the prostate.

**DOI:** 10.1038/bjc.1974.176

**Published:** 1974-08

**Authors:** K. Griffiths


**HORMONES AND THE PROSTATE.** K. Griffiths. Tenovus Institute for Cancer Research, Welsh National School of Medicine, Cardiff.

Regression of prostatic carcinoma in a large proportion of patients treated by anti-androgen therapy, either castration or oestrogen administration, clearly indicates the sensitivity of these tumour cells to hormonal stimulation. This treatment results in the decrease in plasma testosterone, and although the effectiveness of the therapy might suggest that this androgen or its prostatic metabolites may have some aetiological role in the development of cancer of the prostate, there is at present little evidence to incriminate any hormonal imbalance. It is of interest, however, that McNeal (1970, Proc. 3rd Tenovus Workshop, p. 23) has recently challenged the traditional view that carcinoma of the prostate orginates from atrophic, androgen deprived epithelium of the gland in the elderly man. Instead, he suggests that carcinoma arises from active glandular epithelium, again directing attention to an aetiological role of prolonged androgenic stimulation.

It is obvious therefore that further studies are required of the relationship between prostatic disease and endocrine function. Investigations from these laboratories have to date failed to show any major significant differences in the levels of prolactin, LH, FSH, androstenedione, testosterone or oestradiol-17$\beta$ in plasma from normal men and those with either benign prostatic hyperplasia or carcinoma of the gland. More detailed studies in which plasma hormone levels in patients on oestrogen therapy were carefully monitored indicated differences in the endocrine response between patients and suggested that such studies may provide more relevant and interesting data.

For example, the prolactin levels in the plasma were found to be markedly elevated in certain patients after initiation of oestrogen therapy. Many earlier studies have directed attention to the possible relationship between prolactin and the prostate, and it is also known that hypophysectomy can still produce regression of prostatic carcinoma even after orchidectomy and adrenalectomy (Fergusson, 1972, in *Endocrine Therapy in Malignant Disease*, p. 237).

The eventual lack of responsiveness to oestrogen therapy in many patients, resulting in progression of the disease, has been considered due to secondary production of androgen by the adrenal. A series of Synacthen stimulation tests on patients before and during diethylstilboestrol therapy indicated an increased capacity of the adrenal to produce testosterone during treatment.

Little information exists on human prostatic steroid receptor complexes. Such steroid receptor studies in relation to hormone action within the prostatic cell are particularly valuable, and may eventually indicate the mechanism by which oestrogens can directly influence the metabolism of the prostate.